# Incorporating research evidence into decision-making processes: researcher and decision-maker perceptions from five low- and middle-income countries

**DOI:** 10.1186/s12961-015-0059-y

**Published:** 2015-11-30

**Authors:** Zubin Shroff, Bhupinder Aulakh, Lucy Gilson, Irene A. Agyepong, Fadi El-Jardali, Abdul Ghaffar

**Affiliations:** Alliance for Health Policy and Systems Research, World Health Organization, Geneva, Switzerland; Health Economics Unit, Health Policy and Systems Division, School of Public Health and Family Medicine, University of Cape Town, Cape Town, South Africa; Department of Global Health and Development, London School of Hygiene and Tropical Medicine, London, United Kingdom; University of Ghana School of Public Health/Ghana Health Service, Accra, Ghana; Faculty of Health Sciences, American University of Beirut, Beirut, Lebanon

**Keywords:** Evidence, Jacobson’s framework, Low- and middle-income countries, Policymaking

## Abstract

**Background:**

The ‘Sponsoring National Processes for Evidence-Informed Policy Making in the Health Sector of Developing Countries’ program was launched by the Alliance for Health Policy and Systems Research, WHO, in July 2008. The program aimed to catalyse the use of evidence generated through health policy and systems research in policymaking processes through (1) promoting researchers and policy advocates to present their evidence in a manner that is easy for policymakers to understand and use, (2) creating mechanisms to spur the demand for and application of research evidence in policymaking, and (3) increased interaction between researchers, policy advocates, and policymakers. Grants ran for three years and five projects were supported in Argentina, Bangladesh, Cameroon, Nigeria and Zambia. This paper seeks to understand why projects in some settings were perceived by the key stakeholders involved to have made progress towards their goals, whereas others were perceived to have not done so well. Additionally, by comparing experiences across five countries, we seek to illustrate general learnings to inform future evidence-to-policy efforts in low- and middle-income countries.

**Methods:**

We adopted the theory of knowledge translation developed by Jacobson et al. (*J Health Serv Res Policy* 8(2):94–9, 2003) as a framing device to reflect on project experiences across the five cases. Using data from the projects’ external evaluation reports, which included information from semi-structured interviews and quantitative evaluation surveys of those involved in projects, and supplemented by information from the projects’ individual technical reports, we applied the theoretical framework with a partially grounded approach to analyse each of the cases and make comparisons.

**Results and conclusion:**

There was wide variation across projects in the type of activities carried out as well as their intensity. Based on our findings, we can conclude that projects perceived as having made progress towards their goals were characterized by the coming together of a number of domains identified by the theory. The domains of Jacobson’s theoretical framework, initially developed for high-income settings, are of relevance to the low- and middle-income country context, but may need modification to be fully applicable to these settings. Specifically, the relative fragility of institutions and the concomitantly more significant role of individual leaders point to the need to look at leadership as an additional domain influencing the evidence-to-policy process.

## Background

A heightened awareness of the mismatch between our ever increasing wants and scarce resources has brought to the forefront questions of resource allocation and priority setting, and the need to justify decisions taken at every level of the health system. This is all the more important in low- and middle-income countries (LMICs) where there are major health challenges accompanied by limited resources to address these. It is against this background that the use of evidence-based and evidence-informed decision-making have gained ground, both in the field of medicine and, more recently, in health policymaking [1].

However, clinical decision-making is a fundamentally different process from policymaking. Clinical medicine, steeped in the positivist paradigm, seeks evidence of generalizable cause and effect relationships, which are universally applicable as the basis of decision-making. However, evidence is only one of the many inputs that policymakers consider. Policymaking is complex and context dependent, influenced by ethical values, interest group and party politics, as well as social and economic factors. Recognizing this complexity, evidence-informed policymaking has been described as an approach that seeks to ‘ensure that decision-making is influenced by the best available research evidence’, even while it acknowledges the multiplicity of factors that influence policymaking [2].

This paper reflects on the practical experience of a multi-country program (the Sponsoring National Processes for Evidence-Informed Policy Making in the Health Sector of Developing Countries (SNP) programme) aimed at enhancing evidence-informed policymaking in five LMICs supported by the Alliance for Health Policy and Systems Research (AHPSR), an international partnership based within WHO. Over the past decade, WHO has played an important role in promoting evidence-informed policymaking, from the 2004 Ministerial Summit on Health Research, held in Mexico [3], to the 2008 Bamako Call to Action on Research for Health [4] and, most recently, the 2012 WHO Strategy on Health Policy and Systems Research [5].

The approaches used in this programme included (1) encouraging researchers and policy advocates to present their evidence succinctly and vividly, (2) increasing interaction between researchers, policy advocates and policymakers, and (3) strengthening the capacity of Ministries of Health (MOHs) to demand and utilize evidence. While reflections on single projects as well as on specific components of this program have been previously published [6-9], this paper seeks to explore the combination of factors that together explain why projects in some settings were perceived to have moved towards their stated objectives, whereas others were perceived to have not done so well. Additionally, by comparing experiences across five countries, we seek to illustrate general learnings to inform future evidence-to-policy efforts in LMICs.

There are a number of models that seek to explain the incorporation of research evidence into decision-making. These include (1) ‘push’ models, which emphasize the role of producers of research to provide information to decision-makers, (2) ‘pull models’, which give primacy to efforts by research users such as decision-makers to ‘extract information’ from the world of research, (3) ‘exchange models’, which prioritize the creation of linkages or partnerships between producers and users of research, and (4) integrated approaches combining elements of the three aforementioned approaches that make use of knowledge translation platforms that bring together research consumers and producers and encourage both push and pull efforts towards clearly defined goals, often established through priority setting mechanisms [10]. Given, the multi-pronged strategy envisioned by the program and the inevitable variability in project experiences, we use a comprehensive and integrating framework to frame, consider and reflect on these experiences. We believe that the cross-case analysis of these five projects enables us to compare perceived facilitators and hindrances in the evidence-to-policy process in these settings in order to enhance our understanding of evidence-informed policymaking in LMICs.

There exists a significant literature largely centred around high-income countries that examines the use of research evidence in the health policymaking process in an attempt to identify factors influencing the incorporation of evidence into decision-making. These include systematic reviews by Innvaer et al. [11] and by Orton et al. [12]. Other notable studies in high-income countries include those by Harries et al. [13] on evidence-based policymaking in the United Kingdom’s National Health Service, Macintyre et al.’s [14] work on the use of evidence in health policymaking in the United Kingdom, a paper by Lavis et al. [15] examining the role of health services research in policymaking in Canada, and Jewell and Bero’s [16] paper on evidence-informed health policymaking at the State level in the United States.

There is also a growing literature in this area from LMICs. Evidence-informed decision-making faces additional hurdles in these settings. Reflecting funding availability and institutional research capacity, there is often a paucity of locally relevant research evidence available to inform decision-making. This is often accompanied by a shortage of skilled human resources in MOHs to demand, evaluate, synthesize and adapt available research evidence. Frequent transfers of officials within MOHs, something that is not unusual in these settings, further exacerbates this problem. Examples of studies based in LMICs include a literature review and case study on Thailand by Sauerborn et al. [17], a study by Varkevisser et al. [18] based on their reflections on a project to develop national level capacity to use evidence in decision-making, and a study by Pappaionaou et al. [19] based on the experiences of a project designed to encourage the use of data in public health decision-making. These studies identified strong and visionary leadership skills in the use and interpretation of data and the continual engagement of policymakers from the phase of project design and inception as important facilitators, and the frequent turnover of senior officials within the MOH as a significant barrier to incorporating evidence into policy [17-19]. More recent studies include those by Hyder et al. [20], that examine policymakers’ attitudes towards the use and impact of health research in six countries and a study by Cheung et al. [21], who conducted a print media analysis across 44 countries to identify articles exploring health policy priorities, health research evidence, and policy dialogues that could enable evidence-informed health policies. However, there is a need for theoretically-informed cross country comparative research that can explore the combination of factors that enable evidence-informed decision-making in the health systems of LMICs. We address this gap in the literature.

The remainder of this paper is divided into five parts. We begin with a description of projects carried out under the AHPSR’s SNP program (http://www.who.int/alliance-hpsr/projects/national_processes/en/). The second section discusses our preferred theoretical framework, whereas the third describes data sources and methods used to analyse the data. Section four presents the findings and examines these in terms of the theoretical framework. The final section discusses the implications of our study in terms of lessons learned for evidence-informed policymaking for health systems in LMICs.

### The Sponsoring National Processes (SNP) program

The SNP program was launched in July 2008. The program aimed to catalyse the use of evidence generated through health policy and systems research in the policymaking process through (1) promoting researchers and policy advocates to present their evidence in a manner that is easy for policymakers to understand and use, (2) creating mechanisms to spur the demand for and application of research evidence in policymaking, and (3) increased interaction between researchers, policy advocates and policymakers. A number of strategies were suggested including the creation of platforms to produce and communicate research to policymakers in an accessible manner, training policymakers and establishing units within MOHs to strengthen MOH capacity to demand and use research evidence, developing policy briefs (defined as documents that bring together the best available evidence on a problem and viable solutions to address the problem) [22], and supporting fora including conferences and workshops to enable increased interaction between researchers and policymakers to discuss problems, options for addressing these problems and factors to be considered during implementation (policy dialogues) [8,22,23]. However, the decision to implement a particular strategy was left to each individual project. The program also aimed to understand the factors that influenced the use of evidence in policymaking with a view to identify effective strategies to facilitate incorporation of research evidence into policymaking [24]. Grants ran for a three year period. Five projects were supported, based in Argentina, Bangladesh, Cameroon, Nigeria and Zambia [25]. Table 1 provides an overview of these projects.

**Table 1:** **Overview of projects under the Sponsoring National Processes for Evidence-Informed Policy Making in the Health Sector of Developing Countries program**

**Country**	**Name of institution**	**Nature of institution**
Argentina	Centre for the Implementation of Public Policies Promoting Equity and Growth (CIPPEC)	Non-governmental organization
Bangladesh	International Centre for Diarrhoeal Disease Research, Bangladesh (ICDDR,B)	Research and academic institution
Cameroon	Centre for the Development of Best Practices in Health (CDBPH), University of Yaoundé	Research and academic institution
Nigeria	Innovative Health Research Group, Ebonyi State University	Research and academic institution
Zambia	Zambian Forum for Health Research (ZAMFOHR)	Non-governmental organization

The SNP program is thus best viewed as a joint effort involving national stakeholders (including researchers and former policymakers) who designed and implemented individual projects, and an international collaboration, namely the AHPSR, which conceptualized and funded this program as well as mandated the evaluation carried out by a team of international researchers.

### Theoretical framework

We use the theory of knowledge translation developed by Jacobson et al. [26] as a framing device to reflect on our experiences and compare project achievements across the five countries. The framework consists of five domains, namely the ‘user group and its characteristics’, ‘issue under consideration’, ‘available research’, ‘researcher-user relationship’, and ‘dissemination strategies’, each of which contains a number of questions addressing factors that influence the incorporation of evidence into decision-making. This framework takes into account a diverse range of factors and their interactions influencing evidence incorporation into decision-making while providing a tractable analytical framework [26]. Additionally, unlike ‘push’ and ‘pull’ models which view evidence incorporation into policy in terms of unidirectional flows of information, the model acknowledges the role of contextual and political factors. It also goes much further than the ‘two communities’ theory that pessimistically ascribes research non-utilization in policymaking to the intrinsic ‘cultural differences’ between the communities of researchers and policymakers [26,27]. The framework also provides a greater degree of specificity than more recent theoretical frameworks such as the Promoting Action on Research Implementation in Health Services framework [28], which employs extremely broad categories such as level and nature of evidence and research context to explain the incorporation of evidence into policymaking [29].

Jacobson et al. [26] give great attention to the role of the user group and its characteristics. Given that research evidence in this situation is primarily aimed at policymakers, we use the term ‘user groups’ interchangeably with policymakers. The framework hypothesizes that user groups that (1) perceive policymaking as a primarily technical as opposed to political activity, (2) are familiar with research methods and terminology, (3) have previously been involved in utilizing research for decision-making, and (4) have a generally positive as opposed to cynical attitude towards the importance and usefulness of research and researchers are more likely to incorporate evidence into decision-making than user groups that do not display these attributes. Attributes of the issue under consideration also influence whether research evidence is considered in decision-making. Policymakers are more likely to make use of research to guide policy for issues perceived to be highly technical than for those where values and ideology are seen to be more important. Policymakers are also likely to take up issues where there is rapid change and incorporate evidence in coming up with policy solutions to address these problems. Available research constitutes the third domain in this framework. Research that is (1) clear and unambiguous, (2) proposes solutions (as opposed to raising more questions), (3) is amenable to policy action, and (4) perceived to be relevant is more likely to find its way into policy, compared to research that goes against the interests of policymakers or is deemed to be politically unfeasible. The fourth domain is that of the researcher-user relationship. The framework maintains that (1) the establishment of a good working relationship and trust between researchers and policymakers early on, (2) previous experience of working with policymakers, (3) stability of the user group (meaning low turnover of policymakers), (4) agreement on clearly defined outcomes, and (5) delineation of responsibilities of both researchers and policymakers of their roles are positively associated with evidence incorporation into decision-making. The final domain consists of dissemination strategies. Factors that positively influence this include providing policymakers with (1) the quantity of information that they regard appropriate, (2) updates and reminders, (3) clear and vividly presented information, and (4) ensuring ongoing access of policymakers to researcher groups [26].

## Methods

A document review of reports produced by the AHPSR SNP program was conducted at the outset. The projects’ formal evaluation report was the main source of data for this paper [24]. This was supplemented by individual country technical reports that were primarily used to validate information from the evaluation report and add information on a specific area that was not discussed in the evaluation report.

The project evaluation report was produced by two AHPSR appointed external researchers who between them carried out the evaluation across the five projects. Country visits were carried out to monitor project progress, review documents and hold interviews with project leaders, team members, and selected decision-makers, as discussed below. Visits were made to all project sites except Argentina, where this was difficult due to logistic reasons. Additionally, five meetings were held with project teams taking advantage of other events including SURE (Supporting the Use of Research Evidence in African health systems project) meetings as well as the McMaster Health Forum policy dialogues [24]. The evaluation report included information from (1) semi-structured interviews, (2) policy brief evaluation surveys, (3) policy dialogue evaluation surveys, and (4) outcome evaluation surveys. Semi-structured interviews were carried out for all the projects (Table 2). Data from the policy brief and policy dialogue evaluation surveys was available for the Cameroon, Nigeria, and Zambia projects (Tables 3 and 4). However, data for the outcomes evaluation survey was available only for the Nigeria and Zambia projects (Table 5) [24].

**Table 2:** **Sources of data for each project**

**Country**	**Project technical report**	**Semi structured interviews**	**Policy brief evaluation survey**	**Policy dialogue evaluation survey**	**Outcomes evaluation survey**
Argentina	Yes	Yes	No	No	No
Bangladesh	Yes	Yes	No^a^	No^a^	No^a^
Cameroon	Yes	Yes	Yes (n = 78)	Yes (n = 48)	No
Nigeria	Yes	Yes	Yes (n = 43)	Yes (n = 48)	Yes (n = 66)
Zambia	Yes	Yes	Yes (n = 46)	Yes (n = 44)	Yes (n = 48)

^a^The Bangladesh team did not follow the standardized evaluation survey but did its own review which it has termed ‘merit review’.

**Table 3:** **Policy brief evaluation survey**

**Question**	**Cameroon (n = 78), percentage responding very helpful** ^**a**^	**Nigeria (n = 43), percentage responding very helpful**	**Zambia (n = 46), percentage responding very helpful**
	**(Mean score)**	**(Mean score)**	**(Mean score)**
The policy brief described the context for the issue being addressed	90% (6.3)	93% (6.5)	94% (6.6)
The policy brief described different features of the problem, including how it affects particular groups	86% (6.1)	97% (6.5)	94% (6.5)
The policy brief described three options for addressing the problem	88% (6.0)	94% (6.5)	89% (6.3)
The policy brief described what is known, based on synthesized research evidence, about each of the three options and where there are gaps in what is known	80% (6.0)	84% (6.2)	93% (6.5)
The policy brief described key implementation considerations	82% (6.1)	95% (6.5)	89% (6.5)
The policy brief employed systematic and transparent methods to identify, select and assess synthesized research evidence	77% (6.0)	87% (6.5)	82% (6.2)
The policy brief took quality considerations into account when discussing the research evidence	83% (6.1)	91% (6.5)	78% (6.3)
The policy brief took local applicability considerations into account when discussing the research evidence	78% (6.0)	91% (6.5)	82% (6.3)
The policy brief took equity considerations into account when discussing the research evidence	83% (6.2)	85% (6.2)	79% (6.3)
The policy brief did not conclude with particular recommendations	50% (5.4)	54% (5.5)	56% (5.6)
The policy brief employed a graded-entry format	89% (6.4)	83% (6.2)	88% (6.5)
The policy brief included a reference list for those who wanted to read more about a particular systematic review or research study	90% (6.4)	93% (6.7)	94% (6.7)
The policy brief was subjected to a review by at least one policymaker, one stakeholder and at least one researcher	88% (6.3)	86% (6.3)	86% (6.3)
The purpose of the policy brief was to present the available research evidence on a high-priority policy issue in order to inform a policy dialogue where research evidence would be just one input to the discussion^b^		87% (6.2)	88% (6.2)

^a^All questions were on a Likert scale of 1–7 with 1 ‘Very Unhelpful’ to 7 ‘Very Helpful’. Percentage is the total of those responding 6 and 7.

^b^For this question the Likert scale as 1 ‘Failed’ to 7 ‘Achieved’. Percentage is total of those responding 6 and 7.

**Table 4:** **Policy dialogue evaluation survey**

**Question**	**Cameroon (n = 48), percentage responding very helpful** ^**a**^	**Nigeria (n = 48), percentage responding very helpful**	**Zambia (n = 44), percentage responding very helpful**
	**(Mean score)**	**(Mean score)**	**(Mean score)**
The policy dialogue addressed a high-priority issue	92% (6.6)	96% (6.6)	93% (6.5)
The policy dialogue provided an opportunity to discuss different features of the problem, including how it affects particular groups	89% (6.4)	94% (6.6)	93% (6.6)
The policy dialogue provided an opportunity to discuss three options for addressing the problem	94% (6.2)	87% (6.3)	95% (6.3)
The policy dialogue provided an opportunity to discuss key implementation considerations	91% (6.2)	98% (6.6)	88% (6.3)
The policy dialogue provided an opportunity to discuss who might do what differently	89% (6.4)	85% (6.4)	82% (6.1)
The policy dialogue was informed by a pre-circulated policy brief	78% (6.0)	88% (6.5)	83% (6.5)
The policy dialogue was informed by discussion about the full range of factors that can inform how to approach a problem, possible options for addressing it, and key implementation considerations	90% (6.3)	88% (6.5)	87% (6.3)
The policy dialogue brought together many parties who could be involved in or affected by future decisions related to the issue	87% (6.3)	92% (6.6)	90% (6.4)
The policy dialogue aimed for fair representation among policymakers, stakeholders and researchers	90% (6.3)	89% (6.5)	85% (6.4)
The policy dialogue engaged a facilitator to assist with the deliberations	85% (6.3)	94% (6.7)	93% (6.6)
The policy dialogue allowed for frank, off the record deliberations by following the Chatham House rule: ‘Participants are free to use the information received during the meeting, but neither the identity nor the affiliation of the speaker, nor that of any other participant may be revealed’	87% (6.5)	74% (6.2)	86% (6.4)
The policy dialogue did not aim for consensus	83% (6.3)	51% (5.3)	59% (5.7)
The purpose of the policy dialogue was to support a full discussion of relevant considerations about a high-priority policy issue in order to inform action^b^		88% (6.3)	85% (6.3)
		**Percentage responding strongly agree**	**Percentage responding strongly agree**
		**(Mean score)** ^**c**^	**(Mean score)**
I expect to use research evidence of the type that was discussed at the policy dialogue to help work through what I will say in a briefing, advocate for, or decide		87% (6.3)	86% (6.2)
I want to use research evidence of the type that was discussed at the policy dialogue to help work through what I will say in a briefing, advocate for, or decide		98% (6.5)	86% (6.2)
Using research evidence of the type that was discussed at the policy dialogue to help work through what I will say in a briefing, advocate for, or decide is very beneficial		94% (6.6)	97% (6.6)

^a^All questions were on a Likert scale of 1–7 with 1 ‘Very Unhelpful’ to 7 ‘Very Helpful’. Percentage is total of those responding 6 and 7.

^b^For this question the Likert scale as 1 ‘Failed’ to 7 ‘Achieved’. Percentage is total of those responding 6 and 7.

^c^All questions were on a Likert scale of 1–7 with 1 ‘Strongly Disagree’ to 7 ‘Strongly Agree’. Percentage is total of those responding 6 and 7.

**Table 5:** **Outcomes evaluation survey**

**Attribute**	**Nigeria (n = 66)**	**Zambia (n = 48)**
	**Mean score (out of 7)**	**Mean score (out of 7)**
**How often was relevant research evidence about high-priority policy issues easily available to policymakers?** ^**a**^		
Copies of articles or reports about primary research on high-priority policy issues were widely disseminated to policymakers working on these issues	4.3	3.2
Systematic reviews of the research literature on high-priority policy issues were widely disseminated to policymakers working on these issues	4.2	3.1
Policy briefs that described research evidence about a high-priority problem, options for addressing the problem and key implementation considerations were widely disseminated to policymakers working on these issues	4.5	3.5
Policymakers had access to a personal computer with a functional internet connection	3.7	4.4
Policymakers had access to research evidence on high-priority policy issues through a searchable database focused on these issues	3.9	4.1
Policymakers had access to research evidence on high-priority policy issues through a service operated by researchers and designed to respond in a timely way to questions about these issues	3.9	3.3
Research evidence concerning high-priority policy issues was available	4.5	3.8
The research evidence available to policymakers yielded information that could help them address high-priority policy issues	4.6	3.8
**How often did policymakers and researchers interact in the following ways?**		
Policymakers interacted with researchers as part of a priority setting process to identify high priority policy issues for which primary research and systematic reviews were needed	3.9	3.2
Policymakers interacted with researchers as part of the process of conducting primary research or systematic reviews about high-priority policy issues	4.1	3.1
Policymakers interacted with researchers to obtain assistance with finding and using research evidence about high-priority policy issues	4.1	3.2
Policymakers interacted with researchers through targeted efforts to support research use in policymaking	4.0	3.3
Policymakers interacted with researchers on an informal basis (i.e. through membership of committees, personal conversations)	4.4	3.6
**How often did policymakers develop and demonstrate their capacity to find and use health research evidence in health systems policymaking?**		
Policymakers participated in training to develop their capacity to find and use research evidence about high-priority policy issues	4.6	3.1
Policymakers acquired research evidence on high-priority policy issues	4.4	3.5
Policymakers assessed the quality and local applicability of research evidence on high-priority policy issues	4.3	3.4
Policymakers conveyed research evidence on high-priority policy issues to stakeholders in a useful way	4.6	3.4
Policymakers identified or created place for research evidence in decision-making processes	4.4	3.4
**To what extent do you agree or disagree with these statements about the Knowledge Translation (KT) platforms contributions over the last 2 years** ^**b**^		
The KT platform has contributed to enhancing the availability of relevant research evidence on high-priority issues	5.7	5.4
The KT platform has contributed to strengthening relationships among policymakers and researchers	5.6	5.4
The KT platform has contributed to strengthening policymakers capacity to find and use research evidence in health systems policymaking	5.8	5.2

^a^All questions were on a Likert scale of 1–7 with 7 ‘Very Often’.

^b^All questions were on a Likert scale of 1–7 with 7 to a ‘Very Great Extent’.

Semi-structured interview guides were developed by the Knowledge Translation Platform Evaluation Team at McMaster University [24]. Initially, project leaders were interviewed and they in turn identified individuals including policymakers, researchers and members of civil society groups who could serve as key informants. A total of 22 respondents were interviewed across the five project sites. Interviews were conducted face-to-face and were tape recorded; where this was not possible or feasible detailed interview notes were taken [8]. The interviews sought to gather information on a wide range of topics including project activities to create policy-relevant knowledge, activities to generate and strengthen policymaker capacity to demand and use evidence, as well as activities to facilitate interactions among researchers and policymakers. They also provided data on perceptions of project achievements and impact, as well as factors that facilitated or hindered project activities [24].

The evaluation report also included results from surveys on (1) policy briefs produced, (2) policy dialogues held, and (3) project outcomes. Potential respondents for these surveys were identified by project teams based on their likely involvement in policymaking around issues that were being examined by the project teams. Respondents included national and sub-national level policymakers, managers at healthcare institutions, staff members of donor agencies, non-governmental organizations, or professional health associations and researchers at universities and other research institutions [9,24].

For the policy brief survey, identified individuals were posted a package containing policy briefs prepared by the country projects and an invitation to take part in a policy dialogue. Along with the brief and invitation, respondents were provided with a questionnaire to assess the policy briefs that they were required to complete prior to attending the policy dialogue. The questionnaire included questions on respondent perceptions of the process by which the brief was designed and developed as well as an overall assessment of the policy brief itself examined through a 7-point Likert scale [9,24]. A total of 264 individuals were sent the questionnaire which was completed by 167 individuals, with a response rate of over 63%, including 78 respondents from Cameroon, 46 from Zambia, and 43 from Nigeria [9,24].

Immediately after the policy dialogue, participants were provided with a questionnaire to assess the policy dialogue process and were required to complete and return the questionnaire on the spot [9]. The questionnaire was similar in design to that used to assess the policy briefs. With 140 responses out of 237 individuals handed these questionnaires, the response rate for this survey was just under 60% [9].

Finally, for both the Nigeria and Zambia projects, the evaluation report included results from an outcomes evaluation survey conducted after more than 2 years of project implementation to examine views of these same groups of stakeholders on (1) research availability for issues with high priority in health policy, (2) relationships between researchers, government officials and other policymakers, and (3) the capacity of both researchers and policymakers to promote evidence-informed policymaking in the health sector. The outcomes evaluation survey contained a mix of questions, some of which used a 7-point Likert scale while others were open ended questions. The target sample size was up to 50 respondents in each setting, the final sample size was 66 respondents in Nigeria and 48 respondents in Zambia [24].

Information from the evaluation report was supplemented by findings from individual final project technical reports submitted by project teams to the AHPSR [30-32]. These reports typically provided details of activities carried out under the projects including information on the policy briefs developed, policy dialogues conducted and dissemination activities carried out. The project technical report from Bangladesh included findings from the project outcomes evaluation survey, which were based on a tool that was modified from the outcomes evaluation survey in the evaluation report and information on the research teams’ own perceptions of project achievements. The latter information was also included in the final technical report received from the team based in Argentina [30,31].

Using the sources above, we first summarized information on each project in terms of outputs and activities, respondents’ perceptions of policy briefs and policy dialogues, and their perceptions of outcomes, enabling us to categorize projects according to their performance in (1) producing policy relevant evidence, (2) fostering interactions between policymakers and researchers, and (3) building capacity of policymakers to demand and use research.

We then applied the theoretical framework within a partially grounded approach to analyse each of the five cases. This involved coding the data from each of the five projects in terms of the chosen framework as well as allowing for new categories to emerge from the data in order to not force the data to fit the theory [33]. We employed a cross-case analytical approach, drawing on the five projects to provide us insights to draw conclusions that would be generalizable across the cases studied [34,35]. This approach is also widely used for both testing and refining theories, as well as building new theories [36]. Through this process we sought to identify the role of individual variables from our framework, interactions between variables, as well as additional variables emerging from the data.

## Results

Information on project areas and activities is provided in Table 6. Information on respondent perceptions of policy briefs, policy dialogues, and outcomes is provided in Tables 3, 4 and 5, respectively.

**Table 6:** **Project areas and summary of activities**

**Country**	**Project principal focus areas**	**Activities**
Argentina	1. Health insurance reform in Salta province to reduce health system segmentation	• Eight policy briefs
	2. Human resources for health, including distributional issues, health service delivery and incentive structures	• Four policy dialogues
Bangladesh	1. Recommendations for the administration of H1N1 vaccine	• Four policy briefs
	2. Strengthening public sector commitment to addressing non-communicable diseases	• No policy dialogues
	3. Establishment of regulation and a comprehensive policy to address dual practice by public sector health workers	
	4. Creation of mechanisms to ensure the provision of health services to urban street dwellers	
Cameroon	1. Scale up of enrolment in Community Based Health Insurance Schemes	• Five policy briefs
	2. Improving health system governance and facilitating the development of health districts in the country	• Four policy dialogues
	3. Improving the health information system	• Six workshops to build capacity for 42 policymakers and two researchers
	4. Scaling up efforts to control malaria	• Directory of institutions, researchers and other health policy stakeholders created
		• Online clearinghouse developed to provide access to summaries and policy briefs
		• Consultative process to facilitate use of evidence by decision-makers and identification of health policy and systems research priorities
Nigeria	1. Using health information and evidence in the day to day functioning of the health system	• Two policy briefs
	2. Improvements in health systems governance structures	• Two policy dialogues
	3. Health financing reform to improve access, availability and efficiency	• Six workshops to train 92 policymakers
	4. Health service delivery improvements in terms of quality and access	• Formation of Health Policy Advisory Committee to produce policy briefs and conduct policy dialogues
	5. Addressing issues relating to the distribution and performance of health workers	• Executive Training Program in health policy
	6. Expanding the availability of and access to essential medicines, medical technology and equipment in Nigeria	
Zambia	1. Strengthening the health system for the delivery of mental health care	• Three policy briefs
	2. Prevention of post-partum haemorrhage in the community	• Three policy dialogues
	3. Addressing the human resource crisis in the health sector	• Formation of research action group to develop policy briefs

The research team in Argentina appears to have focused its attention solely on the production of policy briefs and holding policy dialogues with little evidence of sustained advocacy and dissemination efforts. There was no information on the impact of the policy briefs or dialogues on the policymaking process nor any evidence of the creation of structural-links across researchers and policymakers or of capacity-creation or strengthening efforts [24,25,30].

In Bangladesh, policy briefs regarding the use of the H1N1 vaccine and non-communicable diseases were assessed to have “*had a huge impact on specific decisions taken by policymakers at the national level*” by the project leader during the semi-structured interview [24,31]. The brief on strengthening public sector commitment to addressing non-communicable diseases was reported by the project’s final technical report as well received by policymakers and development partners as a ‘timely’ document. Of the individuals interviewed as part of the project outcomes evaluation (n = 15), 73% were of the opinion that the project had increased access to research evidence for important health issues in the country in addition to cementing relationships across the research and policymaking communities [31]. There was no evidence of the establishment of any formal mechanism to bring together researchers and policymakers as well as little evidence of the project’s role in the creation of policymaker capacity to increase the use of evidence [25,31].

In the Zambia case, while the impact of the policy briefs on policymaking itself remains unclear, the great majority of respondents felt that the policy briefs and dialogue both achieved their desired objectives (Tables 3 and 4). The project aimed to generate capacity through the organization of Research to Action Groups, comprised of policymakers and researchers, among others, to help create the evidence base and conduct priority setting exercises. However, these groups were not institutionalized and ended their activities with the project’s completion [24,25].

Two of the policy briefs produced by the Cameroon project, on community health insurance and on scaling up malaria control in the country, were asserted by the project leader to have influenced national policies in these areas [24]. Respondent perceptions based on the evaluation surveys were largely positive (Tables 3 and 4). Other activities included the creation of a directory of institutions, researchers and other stakeholders in the health policy field, training workshops in skills such as priority setting and the development of policy briefs, and the establishment of a clearinghouse to enable easy access to policy briefs and relevant academic literature [24,32].

Over 85% of respondents felt that the policy briefs and dialogues undertaken as part of the Nigerian project had largely achieved their goals (Tables 4 and 5). The project evaluation underscored the role of the team’s visionary leadership that facilitated building links with key stakeholders, including the State Minister of Health, who was directly involved in the identification of important priority areas [24]. Activities to strengthen capacity included (1) a series of workshops for policymakers that emphasized the creation of relevant skill sets in areas that respondents identified as constraints to evidence-informed policymaking, (2) the creation of a Health Policy Advisory Committee (HPAC) bringing together officials from the MOH, researchers and civil society representatives to produce policy briefs and dialogues and provide policy advice to the MOH, and (3) designing and conducting an Executive Training Program on evidence-informed policymaking for state policymakers [25]. The project reported that the Nigerian Strategic Health Plan’s stated emphasis on the importance of evidence-informed policies was an example of their impact on the policymaking process in the country [24].

From the above information and Table 6 it is clear that, in terms of their overall performance in (1) producing policy relevant evidence, (2) fostering interactions between policymakers and researchers, and (3) building capacity of policymakers to demand and use research, it appears that the project in Argentina and those in Cameroon and Nigeria lie at opposite ends of the spectrum, with the Bangladesh and Zambia cases providing examples of averagely performing projects.

Next, we used the theoretical framework to frame team experiences and explain variations in project performance. We recognize that the relative importance of each of the five domains is context specific and will vary across settings, and that interactions among the domains are critical. For example, interested user-groups (domain: characteristic of the user-group), are more likely to be concerned about the research produced on the area of work that interests them and would thus tend to commission research that is relevant to them (domain: issue characteristic). They would also tend to make clear to the researcher the type of output that they would find useful in their work (domain: characteristic of dissemination strategy). Conversely, in the presence of an indifferent user group, researchers are more likely to pick issues that they think important and produce outputs that they deem relevant; a situation that is far less likely to lead to evidence incorporation into decision-making. Table 7 summarizes project experiences relevant to each of the domains of Jacobson’s theoretical framework, identifying the potential role of each domain in that project. It also highlights variables identified to be critical to the project achieving its goals in that setting.

**Table 7:** **Theoretical framework applied to five projects**

**Country**	**User group**	**Issue under consideration**	**Available research**	**Researcher-user relationship**	**Dissemination strategies**	**Critical variables**
Argentina	No information	–	+	–	–	
Bangladesh	+	+/−	+	+	+	• Issue under consideration
Cameroon	+	+	+	+	+	• Leadership
Nigeria	+	+	+	+	+	• Leadership
Zambia	+	+	+	+/−	+	• Researcher-user relationship
						• Dissemination strategies

+ facilitator, − barrier.

The findings from Argentina clearly illustrate the importance of the coming together of domains. While we lack much information on user-group perceptions of research and researchers, the project technical report explicitly mentioned the difficulty in getting together experts and policymakers to hold policy dialogues as an obstacle to the projects’ functioning, suggesting a generally low level of policymaker enthusiasm on the topic. Additionally, the groups had never worked together before and there was a frequent turnover of MOH staff, which made the establishment of relationships between researchers and policymakers difficult. The need for the project to actively work to ‘spur political interest’ in the issue of provincial health insurance (the main focus of the researchers’ effort), as mentioned in the project technical report, suggests that this was not an issue that was on the policy agenda at the time [30]. Researchers may thus at times need to pay careful attention to how they frame issues to make policymakers interested in their research. On the basis of the information in the technical report, it would appear that the research team had not thought through their dissemination strategy. While policymakers were sent executive summaries, and policy briefs were published online, researchers did not appear to engage with policymakers about the importance of evidence-informed policymaking on an ongoing basis. This is evident from the observation made in the project’s own technical report, in which the team was unable to comment on how the evidence provided would be used in decision-making [30]. It is not surprising that the grantees reported that the project failed to achieve one of its main goals, namely an agreement with policymakers on the main points for a future health reform agenda.

The experience from Bangladesh, on the other hand, demonstrates how critical perceived relevance of research on a particular issue at a particular instance of time is to explaining the success of the evidence-to-policy process. While we lack quantitative information on user group perceptions, data from the merit review indicates that policymakers were keen to increase their interactions with researchers. In addition to forming the basis of a trusted relationship, the pre-existing links between an established research institution International Centre for Diarrhoeal Disease Research, Bangladesh and health policymakers in Bangladesh facilitated the latter’s early involvement in the project and potentially played an important role in enabling the project to overcome the challenge posed by frequent staff changes in the MOH [31]. It also helped enhance the perceived credibility of the research and the project itself produced research that was perceived as clear and unambiguous by policymakers. Finally, the project was commended by policymakers for its dissemination strategy, which involved preparing two-page policy briefs, and which was decided on after consulting with policymakers [31]. The Bangladesh experience also illustrates the importance of the research issue. The recommendation on the use of H1N1 vaccine, something that was seen as a technical issue in a crisis situation resulted in its incorporation into policy [31]. Few people in the government of Bangladesh had much knowledge of how to deal with the H1N1 crisis and most judiciously use the vaccine, leading them to look for answers [31]. On the other hand, recommendations on the issue of dual practice, seen to be less technical and more administrative, in addition to having controversial implications, did not find their way into policy [26,31].

User groups in Zambia tended to have a positive view of research and researchers, with 97% of respondents mentioning that the use of research evidence was extremely beneficial to their work. An overwhelming 86% of the respondents also felt that they were expected to use research evidence in their work (Table 4). In common with the Nigeria and Cameroon projects, there appeared to be a great deal of overlap of roles between policymakers and researchers [24,26]. The issues that the policy briefs sought to address were decided based on explicit priority setting exercises, and a great majority of the respondents (93%) felt that the policy dialogues (which were on the same topics as the policy briefs) addressed a high-priority issue (Table 4). The available research was well regarded. A total of 89% of respondents felt that the policy briefs were very useful in presenting three options to address the problem (Table 3). The project devised an institutional structure, the Research to Action Group, to bring together researchers and policymakers. The information available on researcher user relationships indicates that the project was not perceived to have performed nearly as well as that in Nigeria (Table 5). Respondents praised the project’s dissemination strategies, in particular the policy briefs, for ease of absorption and enabling quick reading by providing a set of key messages leading up to the main report, with 88% of respondents finding this approach very useful (Table 3). In spite of this, the project did not fully meet the program objectives. The Research to Action groups mentioned above were not institutionalized and ended functioning at the projects completion. Second, despite efforts to engage policymakers and other health systems stakeholders, the extent of dissemination achieved was limited and it appears that the Zambian Forum for Health Research’s mission and mandate remained largely unknown to national policymakers (Table 1) [24].

In common with the information from other African countries, members of user groups in Cameroon appeared to have experience in carrying out research, with 67% of respondents who did not describe themselves as researchers indicating that they had research experience. The project’s activities were facilitated by pre-existing institutional structures, being based at a research institution that had been set up to promote the cause of evidence-informed policymaking in Cameroon’s health sector [24]. This structure headed by an individual formerly associated with the national MOH facilitated a positive researcher-user relationship, extending well beyond the scope of this project, with the research institution playing a significant role in brokering knowledge between researchers and other stakeholders nationally. The issues addressed were deemed to be important, with 92% of respondents reporting that the policy dialogues were very useful in addressing high-priority policy issues (Table 4). The research at hand was well appreciated, with 88% of respondents being of the opinion that the policy briefs were very useful in describing three options for addressing the problem (Table 3). Project dissemination strategies were well developed through the establishment of the online clearinghouse and conducting deliberative forums (on community-based health insurance and malaria control) with policymakers, implementers and research institutions [32]. In terms of clarity and ease of reading, the policy briefs were commended by a majority of respondents, with 89% of respondents finding the provision of a set of key messages leading up to the main report (graded entry format) very useful (Table 3). Other factors that were important included institutional leadership, which brought together the policy and research communities and provided credibility to the evidence-to-policy process, and the establishment of close links between the research institution and the MOH [24,25]. While we recognize that both these factors can be viewed as subsumed under the researcher-user relationship domain, given their role in this project and the Nigerian project as elaborated below it may be wise to independently examine their role.

The Nigeria project followed a similar pattern to Cameroon. User groups wanted to and were expected to use research evidence, with 98% and 87% of respondents, respectively, strongly agreeing with these assertions (Table 4). In common with the Zambia and Cameroon projects, there was a large degree of overlap between the research and policy communities. Among the five projects, this was the only one that was based in a university, though the project was carried out jointly between Ebonyi State University and the MOH. The project brought together researchers and policymakers, aided by the active support provided by the State Minister of Health and institutionalized through the HPAC. The strong researcher-user relationship is reflected by the data on the interaction of these groups in Table 5. The issues taken up were regarded as important, with 96% of the respondents agreeing that the policy dialogue addressed a high-priority issue (Table 4). The research itself was recognized as being useful, with 94% of respondents being of the opinion that the policy briefs were very useful in describing three options to address the issue. Additionally, 95% of respondents felt that the policy briefs did well in describing the most important implementation factors (Table 3). In addition to the HPAC, dissemination was also achieved through radio programs. The project’s emphasis on sensitizing policymakers to the importance of evidence-informed policymaking through workshops and training programs appears to have had positively influenced the receptivity of this group to evidence-informed policymaking [24]. Additional factors that appear to have been important include project leadership, which was perceived as ‘visionary and dedicated’, enabling the project to succeed even when faced with frequent staff changes at the MOH, and the institutional involvement of the MOH which made it much easier to access key decision makers and engage them in the evidence-to-policy process [25].

## Discussion and conclusion

Reflecting on our experiences from these AHPSR-supported projects in five countries across three continents, we can conclude that the successful incorporation of evidence into policymaking is indeed greatly aided by the coming together of a number of domains [19]. The combination of (1) an interested and informed group of policymakers, (2) a research issue that generates sufficient interest in both the research and policymaking communities and that policymakers are seeking immediate solutions for, (3) the availability of methodologically sound research that is easy to understand, (4) good working relationships between researchers and policymakers, and (5) with a clear understanding of expected outcomes and adequate and widespread dissemination of research findings, makes incorporation of research evidence into policymaking more probable. Findings from this paper suggest that strategies that solely focus on providing policymakers with research evidence through policy briefs or policy dialogues are thus usually insufficient in enabling evidence-informed decision-making. Our conclusions are supported by the results of a recent randomized controlled trial by Beynon et al. [37], who found that, though useful, policy briefs alone, without the influence of other factors and interventions did not make much difference in enabling the incorporation of research into policy and practice.

The results of this study are supported by findings from a systematic review by Innvaer et al. [11] that identified personal contact between researchers and policymakers, the timeliness and relevance of research, and the clarity of research in terms of the provision of recommendations as the most important facilitators of the incorporation of evidence into decision-making. Our contentions are also supported by the work of Jewell and Bero [16], as well as by a study by Hyder et al. [20] in six LMICs, which cite poor communication and dissemination of research and the lack of technical capacity on the part of policymakers as significant barriers to using evidence in decision-making.

However, we caution against an understanding of the evidence-to-policy process as a simple coming together of domains. First, as explained above, the domains have the potential to reinforce each other and reflect an interactive spectrum where the whole can be greater or lesser than the sum of the parts. The Argentinian case of apparent user group indifference, apparently weaker pre-existing user group-researcher relationships, and lack of issue salience lies at one end of the spectrum. The Nigerian project, with political interest and involvement, systematic priority-setting exercises and capacity building initiatives, represents the other. Second, the relative contribution of each of these domains varied greatly across the different projects suggesting the importance of other contextual factors in shaping observed effects. For example, it would seem that, in Bangladesh, the issue, including the urgency surrounding it, its technical nature and potential to affect key interest groups, determined whether or not evidence was considered in policy around that issue. On the other hand, in the Zambian experience, the project’s ability to reach out to policymakers determined whether or not research evidence was considered in policymaking around the issue.

Additionally, in line with Harries et al. [13], we find other factors, including the role of individual leaders, to be important in facilitating the evidence-to-policy process. In both Nigeria and Cameroon, project leadership, by bringing together researchers and policymakers, was identified as being vital to the success of the program. This analysis also suggests that the location of the group undertaking knowledge translation efforts and the strength and permanence of its links to various government organizations can have an important bearing on access to key policymakers. An examination of the projects shows that knowledge translation platforms with strong and long-term links to government entities (for example in Nigeria and Cameroon) tended to be more effective than those where links to the government appear to have not been as institutionalized [24,25].

These findings suggest that, though relevant to a large extent, Jacobson’s theoretical framework, initially developed for high-income settings, may need modification to be applicable to LMICs. The relative fragility of institutions and concomitantly more significant role of individual leaders points to the need to look at leadership as an additional domain influencing the evidence-to-policy process.

The evidence from Nigeria, Zambia and Cameroon (where we have this information) also suggests that the separation between the ‘two communities’ of researchers and policymakers discussed above in the theoretical framework [27] is not as rigid as the framework suggests. A large proportion of the policymakers interviewed had experience in research and a number of the researchers interviewed had taken part in policymaking [24]. We would argue that the ‘two communities’ framework, probably has greater applicability to Western contexts where research communities are large and research is largely carried out in universities, than in settings where policy communities (consisting of both researchers and interested policymakers) in fields such as health tend to consist of relatively few individuals. In these settings, informal relationships and personal interactions potentially play an even more important role in effective incorporation of evidence into policy than the theoretical framework might suggest.

Finally, the findings from this study point to the need for comprehensive mechanisms to facilitate the incorporation of evidence in policymaking and overcome what has been termed as the false dichotomy of the ‘know-do’ gap in health policy and systems research [38]. Embedding of research, or the systematic integration of knowledge generation activities within the core functions of health systems in order to make them a more central feature of decision-making processes, is one such mechanism that is central to WHO’s recent strategy on health policy and systems research. In such a situation, decision-makers and researchers are linked in a system that enables decision-makers to easily access researchers who can readily provide timely and relevant evidence to inform policy design and implementation [5]. A number of initiatives exist as examples of this concept. These have taken the form of research centres within MOHs, such as the National Health Systems Resource Centre in India, as well as the use of mandates and legislation to incorporate health policy and systems research into policymaking, as done in Mexico [5].

There are some limitations to this study. The most significant being that the paper is based primarily on the perceptions of stakeholders including researchers, policymakers, and a few civil society and development partner groups who were in turn identified by the country project teams. It is these perceptions as opposed to well-defined policy outcomes that have been used to assess the role of evidence in informing health policymaking. Additionally, individuals identified by the project teams may feel a need to report positively about project processes and outcomes, which is a potential source of bias in the data. The inclusion of a wider range of stakeholders, such as the media as well as politicians, in the sample would have further strengthened and potentially enhanced the generalizability of the findings, in addition to removing this potential conflict of interest. Second, data availability varied by project. Having identical data sources across all the projects would have enhanced our ability to draw inferences by directly comparing results across sites. Third, the project evaluation was led by a team of two outside researchers from major universities in the Middle East and Latin America. While, on the one hand, this reduces the chance of biased analysis and reporting, on the other, it has the potential to lead to a loss of insider perspective that critical self-evaluation can provide and that an outsider may not be able to access through the interview process. Notwithstanding these limitations, we do believe that this study, by comparing the five countries as cases, has enabled us to at least answer some of the ‘why’ questions behind each project’s achievements, to contribute to further build on the chosen theoretical framework and significantly inform efforts towards evidence-informed health policy in LMICs.

## Abbreviations

AHPSR: Alliance for Health Policy and Systems Research
HPAC: Health Policy Advisory Committee
LMICs: Low- and middle-income countries
MOH: Ministry of Health
SNP programme: Sponsoring National Processes for Evidence-Informed Policy Making in the Health Sector of Developing Countries programme
